# Behçet's Disease Under Microbiotic Surveillance? A Combined Analysis of Two Cohorts of Behçet's Disease Patients

**DOI:** 10.3389/fimmu.2020.01192

**Published:** 2020-06-12

**Authors:** Tim B. van der Houwen, Jan A. M. van Laar, Jasper H. Kappen, Petrus M. van Hagen, Marcel R. de Zoete, Guus H. van Muijlwijk, Roos-Marijn Berbers, Ad C. Fluit, Malbert Rogers, James Groot, C. Marijn Hazelbag, Clarissa Consolandi, Marco Severgnini, Clelia Peano, Mario M. D'Elios, Giacomo Emmi, Helen L. Leavis

**Affiliations:** ^1^Section Clinical Immunology, Departments of Internal Medicine and Immunology, Erasmus University Medical Center Rotterdam, Rotterdam, Netherlands; ^2^Allergy and Clinical Immunology, Immunomodulation and Tolerance Group, Inflammation Repair and Development, Imperial College, National Heart and Lung Institute, London, United Kingdom; ^3^Department of Pulmonology, STZ Centre of Excellence for Asthma and COPD, Franciscus Group, Rotterdam, United Kingdom; ^4^Medical Microbiology, University Medical Center Utrecht, Utrecht, Netherlands; ^5^Department of Rheumatology and Clinical Immunology, University Medical Center Utrecht, Utrecht, Netherlands; ^6^Department of Epidemiology, Julius Center for Health Sciences and Primary Care, University Medical Center Utrecht, Utrecht, Netherlands; ^7^National Research Council, Institute of Biomedical Technologies, Segrate, Italy; ^8^National Research Council, Institute of Genetic and Biomedical Research, UoS Milan, Milan, Italy; ^9^Genomic Unit, Humanitas Clinical and Research Center, Milan, Italy; ^10^Department of Experimental and Clinical Medicine, University of Firenze, Firenze, Italy

**Keywords:** Behçet's disease, microbiota, intestinal, oral, IgA-SEQ

## Abstract

**Background:** In Behçet's disease (BD), an auto-inflammatory vasculitis, an unbalanced gut microbiota can contribute to pro-inflammatory reactions. In separate studies, distinct pro- and anti-inflammatory bacteria associated with BD have been identified.

**Methods:** To establish disease-associated determinants, we performed gut microbiome profiling in BD patients from the Netherlands (*n* = 19) and Italy (*n* = 13), matched healthy controls (HC) from the Netherlands (*n* = 17) and Italy (*n* = 15) and oral microbiome profiling in Dutch BD patients (*n* = 18) and HC (*n* = 15) by 16S rRNA gene sequencing. In addition, we used fecal IgA-SEQ analysis to identify specific IgA coated bacterial taxa in Dutch BD patients (*n* = 13) and HC (*n* = 8).

**Results:** In BD stool samples alpha-diversity was conserved, whereas beta-diversity analysis showed no clustering based on disease, but a significant segregation by country of origin. Yet, a significant decrease of unclassified *Barnesiellaceae* and *Lachnospira* genera was associated with BD patients compared to HC. Subdivided by country, the Italian cohort displays a significant decrease of unclassified *Barnesiellaceae* and *Lachnospira* genera, in the Dutch cohort this decrease is only a trend. Increased IgA-coating of *Bifidobacterium* spp., *Dorea* spp. and *Ruminococcus bromii* species was found in stool from BD patients. Moreover, oral Dutch BD microbiome displayed increased abundance of *Spirochaetaceae* and *Dethiosulfovibrionaceae* families.

**Conclusions:** BD patients show decreased fecal abundance of *Barnesiellaceae* and *Lachnospira* and increased oral abundance of *Spirochaetaceae* and *Dethiosulfovibrionaceae*. In addition, increased fecal IgA coating of *Bifidobacterium, Ruminococcus bromii* and *Dorea* may reflect retention of anti-inflammatory species and neutralization of pathosymbionts in BD, respectively. Additional studies are warranted to relate intestinal microbes with the significance of ethnicity, diet, medication and response with distinct pro- and inflammatory pathways in BD patients.

## Introduction

Behçet's disease (BD) is an auto-inflammatory vasculitis, characterized by mucosal ulcerations, skin lesions and uveitis (1). Immunosuppressives are indicated in organ or vision threatening cases (2). BD seems to be driven by an excessive T-cell reaction that might be triggered by an infectious antigen in a genetically susceptible host (3). This is emphasized in the association with HLA-B51 and non-HLA genetic associations like IL10, IL23R, and ERAP1, which suggests a genetic susceptibility similar to spondyloarthritis (4). Increased salivary colonization with *Streptococcus mutans* has been demonstrated in BD patients and both skin and serum testing with streptococcal antigens induce inflammatory reactions (5–8). Furthermore, antibiotics added to regular treatment has yielded a significant improvement of symptoms in BD patients (9). Subsequently, dysbiosis of the Gut Microbiome (GM) might trigger or exacerbate inflammatory processes in BD patients.

Dysbiotic GM, showing, for example a reduced intestinal microbial diversity, has been observed in BD patients before. Specifically, significant depletion of *Roseburia* and *Subdoligranulum* species leading to decreased butyrate production has been suggested to contribute to inflammation (10). Furthermore, a significant increase of *Bifidobacterium* and *Eggerthella* genera and reduction of *Megamonas* and *Prevotella* genera have been reported in a Japanese BD cohort compared with matched healthy controls (HC) (11). *Rothia dentocariosa*, a known abundant inhabitant of the oral cavity of BD patients, was found decreased in active ulcerations sites. Moreover, oral *Neisseria* and *Veillonella* numbers were decreased in both active and inactive BD (12). These studies demonstrate that different pro- and anti-inflammatory oral and gut bacteria, among different BD populations, may contribute as potential disease making determinants. Furthermore, identification of specific immunoglobulin A-(IgA-) coated bacteria may aid in identification of pro- or anti-inflammatory commensals by IgA-SEQ analysis (13). For instance, in Crohn's disease (CD) and spondylarthritis, a selective enrichment in IgA-coated *Escherichia coli* was found, which induced a mucosal and systemic Th17 response when administered in mice (14). In this observational study on a Dutch cohort, we aimed to extend insights into GM and Oral Microbiome (OM) in BD. We compared this cohort to an Italian one, and performed IgA-SEQ analysis in fecal samples of Dutch BD patients.

## Methods

### Ethics Statement

Ethic approval for this study was received from the Medical Ethical Committee of the Erasmus MC (MEC-2012-220). Written informed consent was obtained from all patients and controls according to the Declaration of Helsinki.

### Study Design

In this observational study, patients were selected at the outpatient clinic of the Erasmus MC in Rotterdam, the Netherlands or the outpatient clinic of the Florence Behçet Center, Florence, Italy. All patients were diagnosed according to the International Study Group of BD criteria (15). Only patients aged 18 years or older were included. Use of proton pump inhibitors or any antibiotics in the 3 months preceding the study was used as exclusion criteria. Healthy controls included family members and non-family members, matched for ethnic background. Inclusion and exclusion criteria for the Italian cohort are the same as those described in Consolandi et al. (10) and are overlapping in the use of antibiotics as exclusion criteria.

### Sample Collection and DNA Extraction

In the Dutch cohort, samples were taken from non-ulcerated tissue around the inferior labial frenulum using oral swabs [Copan swab 482 CE with Amies medium (Murrieta, CA, USA)] and stored at −80°C. Stool samples [Sterilin UK specimen container, Thermo-Fisher (Waltham, Massachusetts, USA)] were gathered at the outpatient clinic or at patients' homes. Samples were transferred on dry ice and stored at −20° C within 24 h, for a maximum of 14 days, when samples were transported to −80°C. DNA was extracted from stool, for both the Dutch and the Italian cohort, with QIAamp DNA Stool Mini Kit (QIAGEN, Valencia, CA, USA) and from oral swabs with AGOWA mag Mini DNA Isolation Kit (AGOWA, Berlin, Germany) as previously described (16, 17).

### 16S rRNA Gene Sequencing

The extracted DNA, for both oral and fecal samples, was sequenced following the protocol described before (18). Briefly, a 469 bp amplicon encompassing the V3 and V4 hypervariable regions of the 16S rRNA gene was amplified and sequenced using the Illumina MiSeq Reagent Kit v3 2 × 300 bp (600-cycle) or the Illumina MiSeq Reagent Kit v2 2 × 250 bp (500-cycle) on a MiSeq Illumina sequencer according to Fadrosh et al. (19). To monitor potential contamination during DNA extraction, amplification and sequencing protocol negative- and buffer controls were used.

### Bioinformatic Data Analysis

In order to have identical read lengths for all samples, reads from the 2 × 300 bp run were trimmed down to 250 bp to match the other dataset. All reads were pre-processed as described (18) and merged using FLASH (version 1.2.11) (20) with options “-t 1” and “-M 167.” Subsequently, merged reads were de-multiplexed using the “split_libraries_fastq.py” script and further analyzed using the QIIME microbial community analysis pipeline (version 1.9.1) (21). Quality filtering was also performed, using the split_library_fastq.py script in qiime, with the following non-default settings: q20 (truncating reads with an average PHRED quality score of 20 or less), max_bad_run_length 50 (trimming reads after 50 following bases with an average PHRED quality score of 20 or less), barcode_type24, max_barcode_errors 3. After removal of the barcodes, heterogeneity spacers, and primer sequences, about 6.2 million sequences were left, with a mean length of 409 bp (median length of 404 bp). Sequences were assigned to operational taxonomic units (OTUs) at 97% similarity using QIIME's open-reference OTU picking workflow (“pick_open_reference_otus.py”) and the USEARCH package (version 6.1.544) (22), in addition to detection and removal of chimeric sequences (which is performed by UCHIIME as part of the USEARCH package). The OTU sequences were aligned to the Greengenes 16S rRNA gene database (13.8 release), followed by removal of OTUs represented by <0.005% of the total number of sequences, which was 5.4 million. The average number of sequences per samples was 73,900 (median 80,100). The generated OTU table was used for assessing alpha- and beta-diversity using QIIME's core_diversity_analyses.py workflow with a rarefaction depth of 10,800 sequences, chosen based on the lowest amount of sequences while maintaining the most amount of samples as possible.

### IgA-SEQ Analysis

IgA-SEQ analysis was performed as described before (13). Briefly, bacteria in fecal samples were stained with PE-conjugated Anti-Human IgA (Miltenyi Biotec clone IS-8E10, Cologne, Germany) prior to fluorescence-activated cell sorting flow cytometric analysis and sorting of IgA positive bacteria. After isolation of bacterial DNA from 200,000 sorted bacteria (both IgA positive and IgA negative), 16S rRNA sequencing of the V4 region was performed on an Illumina miSeq (2 × 250) using barcoded primers (23). IgA Coating Index scores were calculated by dividing the relative abundance of a species in the IgA+ fraction by the relative abundance of that species in the IgA- fraction.

### Statistical Data Analysis

For Principal Component Analysis (PCA), R 3.5.0 within RStudio 1.1.463 (RStudio Team, Boston, MA, USA) (24) was employed, using zCompositions, clr transformation and ggplot R packages (25, 26). Principal Coordinate Analysis (PCoA) with Bray-Curtis distance was used to calculate beta-diversity of the samples, with permutational multivariate analysis of variance (PERMANOVA) to test for statistical significance. Shannon index was used to calculate alpha-diversity with Wilcoxon Rank to test for statistical significance.

Microbial diversity and statistical analyses for IgA-SEQ were performed with QIIME (v. 1.8.0), the Vegan package for R, and LEfSe (21, 27).

Microbiota changes between BD patients, HC and the different cohorts (patients subdivided by country of origin or HLAB51 status) were investigated using ANCOM (28) in R 3.3.3 (24). For statistical testing, we used false discovery rates (FDRs) correction for multiple comparisons and a FDR- adjusted *p*- value < 0.05 was considered significant (29).

## Results

### Patients and Data Characteristics

Patients and HC were included in a Dutch and an Italian cohort. The Dutch cohort consisted of 19 patients [mean age: 50 years (range: 34–65)] and 17 age-, gender- and ethnicity-matched HC; the Italian cohort of 14 patients [mean age: 45 (range: 30–71)] and 15 age-, gender- and ethnicity matched HC. From all patients and HC, stool samples were taken. Oral samples were provided by 18 Dutch patients and 15 Dutch HC.

Although both patient cohorts showed comparable disease manifestations, notable differences in medication usage were present. In the Italian cohort, corticosteroids were significantly (*p* < 0.01) more often prescribed (Table 1).

**Table 1:** Patient characteristics.

	**Dutch cohort**		**Italian cohort**	
	**Patients**	**Controls**	**Patients**	**Controls**
	*n =* 19	*n =* 17	*n =* 13	*n =* 15
Age^*^	50 (34–65)	45	45 (30–71)	44
Female^**^	53	47	54	53
Behçet's disease symptoms^**^				
- oral ulceration	100	–	100	–
- genital ulceration	84	–	69	–
- skin lesions	79	–	85	–
- uveitis	37	–	31	–
- HLAB51 positive	47	–	54	–
Medication use^**^				
- colchicine	74	–	62	–
- biologicals	16	–	38	–
- corticosteroids	5	–	77	–

* Years (mean, range).

** *Expressed as %*.

### Characterization of GM in BD Patients and HC

**Figure 1:**
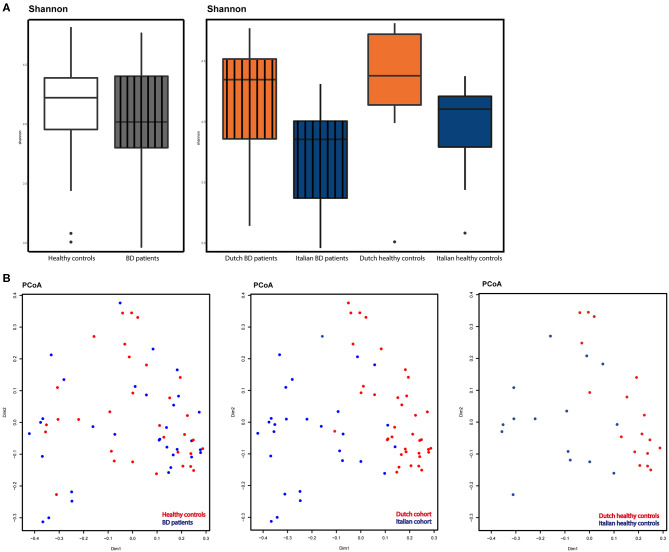
**(A)** Alpha-diversity as displayed by Shannon index. Left: patients (gray, striped) compared to HC (white). Right: alpha-diversity in Dutch BD patients (striped, orange), Italian BD patients (striped, blue), Dutch HC (orange) and Italian HC (blue). **(B)** Beta-diversity as displayed by principal coordinate analysis (PCoA) based on the Bray-Curtis metric. Left: diversity in BD patients (blue) vs. HC (red). Middle: diversity compared between countries, Italy (blue) vs. the Netherlands (red). Right: diversity compared between countries in healthy controls, Italy (blue) vs. the Netherlands (red).

Using a cut-off value defined at 10,800 reads, 64 samples were selected (1 sample excluded because of too few sequence reads) for subsequent analyses. To study microbial alpha-diversity, Shannon index was calculated, showing no significant differences between 32 patients [Netherlands (NL) *n* = 19, Italy (IT) *n* = 13] and 32 HC (NL *n* = 17, IT *n* = 15). The Italian cohort displayed a significant lower alpha-diversity (p=0.006), compared to the Dutch cohort. Moreover, in Italian patients, a trend toward a decreased alpha-diversity was observed compared to their HC (Figures 1A,B).

PCoA demonstrated a similar beta-diversity, with no distinct clustering of patients or HC. A significant (*p* < 0.001) segregation between patients based on country of origin was observed, as also shown in the PCoA plot displaying only healthy controls from both countries (Figure 1B).

ANCOM analysis was used to identify differences in GM between BD patients and HC at taxonomical levels of family and genus.

**Figure 2:**
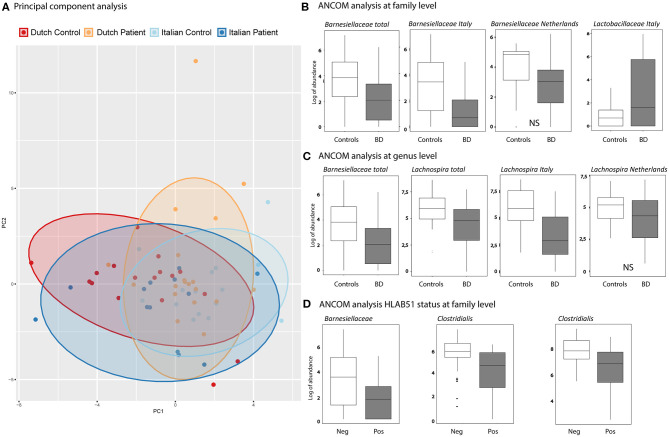
**(A)** Principal components analysis (PCA), based on GM genera abundance, showing Dutch HC (red), Dutch BD patient (orange), Italian HC (light blue), Italian BD patient (dark blue). **(B,C)** Boxplots show the results of ANCOM analysis at family **(B)** and genus **(C)** level. Log of abundance in both healthy controls (white boxes) and BD patients (gray boxes). NS is non-significant. **(D)** ANCOM analysis at family level of HLAB51 positive (gray boxes) and HLAB51 negative patients (white boxes) displayed in log of abundance.

A depletion of the relative abundance of the family *Barnesiellaceae* in BD patients was found compared to HC. In separate analysis of the Italian and Dutch cohorts, only the Italian BD patients displayed a significant decrease in *Barnesiellaceae*. In the Dutch cohort the decrease was observed as a trend. In addition, an increase in the relative abundance of the family of *Lactobacillaceae* was shown for the Italian cohort compared to healthy controls. This was not observed in the Dutch cohort (Figure 2). Analysis at genus level in the combined cohort revealed a significant decrease of relative abundance in unclassified *Barnesiellaceae* and in *Lachnospira* in BD patients compared with HC (Figure 2). This decrease in *Lachnospira* was also significant in separate analysis of Italian BD patients, where in Dutch patients this decrease was only observed as a trend.

Further analysis, subdividing patients into cohorts by clinical parameters, showed a distinct microbiome in HLAB51-positive patients. In these patients, ANCOM analysis displayed a significant decrease in relative abundance of the family of *Barnesiellaceae* and two unclassified families of the *Clostridiales* order (Figure 2). Other subgrouping of patients based on disease manifestations was not contributing.

**Figure 3:**
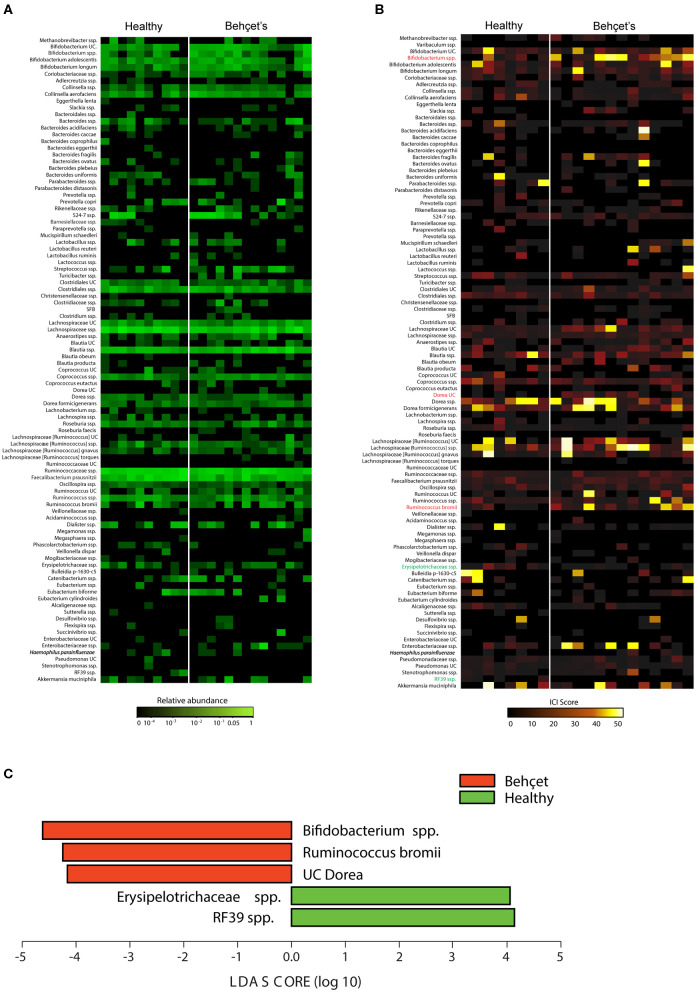
**(A)** Abundance heatmap: heatmap depicting relative abundance of bacterial species in fecal bacteria from BD patients (*n* = 13 samples) and HC (*n* = 8 samples). The relative abundance heatmap is depicted on a logarithmic scale. UC, unclassified in the Greengenes reference database. **(B)** IgA-Coating Index (ICI) heatmap: Heatmap depicting ICI scores of bacterial species in fecal bacteria from BD patients (*n* = 13 samples) and HC (*n* = 8 samples). Species that are significantly enriched in BD patients as compared to HC are depicted in red, species that are significantly IgA-coated in HC as compared to BD patients are depicted in green (LEfSe; *p* < 0.05). UC, unclassified in the Greengenes reference database. **(C)** Lefse plot: LEfSe comparisons of ICI scores of bacterial species from BD patients and HC. Taxa that are significantly enriched in BD patients are depicted in red, and taxa that are significantly enriched in the healthy controls are depicted in green. Significance levels for LEfSe were *p* < 0.05 and Linear Discriminant Analysis (LDA) Score > 2.

On fecal samples from 13 patients and 8 HC of the Dutch cohort, IgA-SEQ analysis was performed and showed distinct IgA coating of taxa in BD and HC, not found by traditional 16S rDNA sequencing. In Figures 3A,B the differences in the heatmaps of traditional 16s rDNA sequencing and IgA-Coating Index are displayed. Three species showed increased IgA coating in BD patients as compared to HC: *Bifidobacterium* spp, *Dorea* spp and *Ruminococcus bromii* spp. (Figure 3C). In contrast, HC showed an increase in IgA inducing *Erysipelotrichaceae spp*. and *RF39 spp*. as compared to BD patients. To clarify, the overall abundance of this species is displayed in Table 2, no significant differences in abundance of this species were observed.

**Table 2:** IgA-SEQ analysis, relative abundance of species with significant differences in IgA coating.

	**Behçet's disease**	**Healthy control**
**Taxonomic units**	**Average relative abundance (standard deviation)**	
***Bifidobacterium*** **spp**.	0.0867 (0.11)	0.0387 (0.03)
***Rominucoccus bromii*** **spp**.	0.0088 (0.01)	0.0065 (0.01)
***UC Dorea*** **spp**.	0.0002 (0.00)	0.0004 (0.00)
***RF39*** **spp**.	0.0001 (0.00)	0.0018 (0.00)
***Erysipelotrichaceae*** **spp**.	0.0074 (0.00)	0.0056 (0.01)

### Oral Microbiome Profiling in Dutch BD Patients

**Figure 4:**
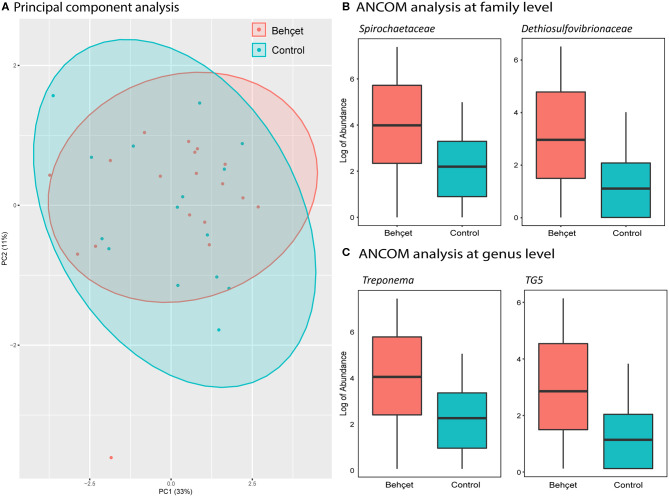
**(A)** Principal components analysis (PCA), based on oral microbiota genera abundance, showing Dutch BD patients (orange) and Dutch HC (light blue). **(B,C)** Boxplots show the log of abundance of bacteria with significant increase in relative abundance (ANCOM analysis) in BD patients on family **(B)** and genus level **(C)**. HC are represented with the light blue boxes; BD patients are shown as orange boxes.

Oral samples of 18 patients were compared with oral samples of 15 matched and HC, all from the Dutch cohort. Alpha-diversity was not significantly different between BD patients and HC. PCA revealed similar β-diversity, with no distinct clustering of patients from HC (Figure 4). By ANCOM analysis, oral samples from patients with BD were enriched in the family of *Spirochaetaceae* and *Dethiosulfovibrionaceae* (Figure 4). Analysis at genus level revealed a significant increase in the relative abundance of both *Treponema* and *TG5* in patients with BD (Figure 4).

## Discussion

In combined Dutch and Italian cohorts, alpha and beta diversity were not different from HC, yet patients with BD demonstrated a reduced fecal abundance of family *Barnesiellaceae*, and of unclassified *Barnesiellaceae* and *Lachnospira* genera. In separate cohort analysis, we demonstrate the Italian cohort drives the observed deviations. In the Dutch cohort the reduced fecal abundance of the family *Barnesiellaceae* and of unclassified *Barnesiellaceae* and *Lachnospira* genera does not reach statistical significance. Next to this, IgA-SEQ analysis revealed an association of BD with the presence of IgA-coated *Bifidobacterium* spp., *Ruminococcus bromii* and *Dorea* spp., and a reduced presence of IgA-coated *Erysipelotrichaceae* spp. and *RF38* spp.

We demonstrated a decrease in abundance of *Barnesiellaceae* at both family and genus level and *Lachnospira* at genus level in patients with BD. Reduced abundance of *Barnesiellaceae* in stool is related with colitis in IL-22 deficient mice (30). Moreover, reduced abundance of *Barnesiellaceae* is inversely correlated with TNF-α levels serum in treated chronic HIV patients. (31) *Barnesiellaceae* might exert a protective anti-inflammatory effect by reducing TNF-α level, one of the key and target cytokines in BD. Decreased abundance could, therefore, be a contributing factor to inflammation in BD. Decreased abundance of *Lachnospira* has not been associated with BD so far. Reduction of *Lachnospira* has been reported in CD with a decrease in production of short-chain fatty acid (SCFA), known to modulate inflammation through Tregs and suppression of the NF?β-pathway (32, 33). Interestingly, production of butyrate is significantly reduced in BD (10). This decreased production of butyrate may be mediated through reduced abundance of *Lachnospira*, thereby contributing to the inflammatory state in BD. In HLAB51 positive patients, decreased abundance of *Barnesiellaceae* and two unclassified families of the *Clostridiales* order, suggests that genetic background may influence the GM. This hypothesis is supported by healthy HLAB27+ siblings of spondyloarthritis patients, which display a distinct GM compared to healthy HLAB27- siblings (34).

In the Dutch cohort, IgA-SEQ analysis revealed increased IgA-coated *Bifidobacterium, Dorea* and *Ruminococcus* species, indicating that these microbes drive specific immunostimulatory responses and might be pathosymbionts in BD (14). Although *Bifidobacterium* has been found enriched in the fecal microbiomes of a Japanese BD cohort (11), a role as pathosymbiont for *Bifidobacterium* seems counterintuitive, since in other inflammatory disorders (such as CD) *Bifidobacterium* is believed to be a probiotic species that exerts immunomodulatory effects (35) Similar to *Bifidobacterium*, a pathogenic role for *Ruminococcus bromii* seems unlikely. This amylolytic bacteria is mainly known for its role in the degradation of starch, which is an essential component for the formation of SCFA, like propionate and butyrate. Both of these SCFAs induces IL10-producing Treg cells and, therefore, *Ruminococcus bromii* is generally considered as anti-inflammatory (36, 37). This is further supported by the decrease in relative abundance observed in CD patients (38, 39). The induced IgA-coating of both *Bifidobacterium* and *Ruminococcus bromii* in BD might, then, serve to enable efficient retention of the bacteria in the gut mucosa, contributing to sustainable homeostasis by dampening pro-inflammatory signaling in the host (40, 41). Nevertheless, a role as pathosymbiont in BD seems more likely for *Dorea*. Increased relative abundance of *Dorea* has been associated with an increment of intestinal permeability, thereby contributing to inflammation in inflammatory bowel diseases (42). The significance of increased IgA-coating of the respective bacteria needs to be further investigated, for instance, in mouse models.

Decreased diversity in microbiota has been found in CD (43), psoriatic arthritis (44) and it is associated with an inflammatory phenotype (45). A decreased diversity in microbiota has also been shown in BD patients (10). The Dutch cohort, in contrary, like the Japanese one (11), shows a comparable Shannon diversity between patients and HC. Analysis of the cohorts from two different countries showed no differences in both alpha- and beta- diversity in fecal microbiome between patients and HC. Differences among samples are evidently more strongly driven by country of origin instead of disease status. This could reflect either country-specific differences, such as diet or corticosteroid use in the Italian cohort, but also, differences in DNA extraction and PCR protocols. Moreover, “batch effect,” due to sequencing Italian and Dutch individuals on different runs, cannot be excluded. Because of these differences, we decided to also perform analysis on the Dutch and Italian cohorts separately, which prevented to take advantage from increased statistical power on analysis on larger combined sample numbers.

Oral dysbiosis may lead to systemic inflammation, as in periodontitis (46). Despite similar alpha-and beta-diversity in the Dutch cohort, we distinguished increased abundance of families *Spirochaetaceae* and *Dethiosulfovibrionaceae* in oral microbiota of BD patients. Increased abundance of *Dethiosulfovibrionaceae* and *Spirochaetaceae* were both associated with periodontitis and endodontic infections, which are more prevalent in patients with severe BD (47–49).

## Conclusions

In conclusion, we demonstrated decreased fecal abundance of *Barnesiellaceae* on family and genus level and of *Lachnospira* on genus level and increased oral abundance of *Spirochaetaceae* and *Dethiosulfovibrionaceae* on family level in patients with BD. The Italian cohort drives the observed deviations, which are only observed as a trend in the Dutch cohort. Next to this, increased fecal IgA-coating of *Bifidobacterium* and *Ruminococcus bromii* may reflect retention of anti-inflammatory species and increased fecal IgA-coating of *Dorea* may identify a pathosymbiont in BD. Additional studies, however, are warranted to relate intestinal microbes with the significance of ethnicity, diet, medication, and response with distinct pro- and inflammatory pathways in BD patients.

## Data Availability Statement

The datasets generated for this study can be found in the ENA database, study accession number PRJEB36332.

## Ethics Statement

The studies involving human participants were reviewed and approved by Medical Ethical Committee of the Erasmus MC. The patients/participants provided their written informed consent to participate in this study.

## Author Contributions

TH, JL, JK, PH, and HL designed the experiment. MZ and GM performed the IgA-SEQ analysis. R-MB, AF, JG, and CH analyzed the data (both cohorts). CC, MS, CP, MD, and GE are responsible for the Italian experiment and collecting of data. All authors read and approved the final manuscript.

## Conflict of Interest

The authors declare that the research was conducted in the absence of any commercial or financial relationships that could be construed as a potential conflict of interest.

## Abbreviations

BD: Behçet's disease
HC: Healthy controls
GM: Gut microbiome
CD: Crohn's disease
Ig: Immunoglobulin
IT: Italy
NL: the Netherlands
OM: Oral microbiome
PCoA: Principle Coordinate Analysis
PCA: Principle Component Analysis
SCFA: Short-chain fatty acid.
